# Re-Introducing the Maintenance Slot in the Maidstone KMPT ECT Suite and Enhancing the ECT Clinic’s Capacity by 20%

**DOI:** 10.1192/bjo.2025.10405

**Published:** 2025-06-20

**Authors:** Maria Moisan, Stanimira Gogova, Chiara Rubino, Brett Metelerkamp

**Affiliations:** Kent and Medway NHS and Social Care Partnership Trust, Maidstone, United Kingdom

## Abstract

**Aims:** Kent and Medway NHS and Social Care Partnership Trust (KMPT) had been providing fewer Electroconvulsive Therapy (ECT) treatments per capita compared with the national average. Following a reduction in patient numbers after the COVID-19 pandemic, the KMPT ECT Suite aimed to offer both initial and maintenance therapy. This required increasing the number of sessions per day to enhance clinic capacity by 20% to accommodate the reintroduction of a maintenance slot.

The project aimed to enhance the ECT clinic’s capacity by 20%, from 6 to 7 patients per session, in order to reintroduce the maintenance slot. The goal was to provide continuity of care, especially for patients who relapse during specific periods, such as the winter or festive seasons.

**Methods:** A quality improvement approach was utilized, starting with a process map and Gemba walk to observe the day-to-day operations of the ECT suite. Both assessments revealed smooth clinic operations with no major issues. Data review showed that 25% of clinics ran at full capacity, suggesting there was room to trial the reintroduction of the maintenance slot with current staffing levels.

The project used the Plan-Do-Study-Act (PDSA) methodology to trial the maintenance slot. The plan involved offering 5 inpatient slots, 1 community slot, and 1 maintenance slot per month. The maintenance slot was trialled once a month, with the aim to reduce readmissions for specific patient groups. The trial ran from October 2023 to March 2024. During this time, the team was able to accommodate 7 patients per session, but increasing the number to 8 proved difficult. However, after an agreement from the Senior Leadership Team (SLT), the clinic extended its hours to accommodate up to 8 patients per session.

**Results:** Results showed that each ECT session, including treatment and recovery, took approximately 39 minutes. Clinics averaged 180 minutes per session, with 5 patients per session, although the clinic capacity was 6. Despite fluctuations in patient numbers, 25% of clinics reached full capacity.

**Conclusion:** Data analysis from the trial period demonstrated that the team could accommodate the increased workload for a short period. Given that 25% of clinics already operated at full capacity, further efforts to increase ECT uptake may require a review to assess the sustainability of the maintenance slot. Feedback from staff confirmed that 7 patients per session could be managed with current staffing, provided transportation was efficient. The team also began recording safety huddle notes, enhancing data-driven decision-making and tracking attendance strictly.

